# The Impact of Childhood Abuse on the Development of Early Maladaptive Schemas and the Expression of Violence in Adolescents

**DOI:** 10.3390/bs15070854

**Published:** 2025-06-24

**Authors:** Cornelia Rada, Alexandra-Elena Neagu, Valentina Marinescu, Anda-Anca Rodideal, Robert-Andrei Lunga

**Affiliations:** 1 Biomedical Department, “Francisc I. Rainer” Institute of Anthropology, Romanian Academy, Romanian Academy House, 13 September Avenue, No. 13, 5th District, 050711 Bucharest, Romania; corneliarada@yahoo.com; 2 Faculty of Sociology and Social Work, University of Bucharest, Schitu Măgureanu Boulevard No. 9, 1st District, 010181 Bucharest, Romania; valentina.marinescu@sas.unibuc.ro; 3 Municipal Centre of Resources and Educational Assistance Bucharest, Heliade Între Vii Street No. 36, 2nd District, 023384 Bucharest, Romania; a_rodideal@yahoo.com; 4 Department of Psychology, “Constantin Rădulescu-Motru” Institute of Philosophy and Psychology–Romanian Academy, School of Advanced Studies of the Romanian Academy, 5th District, 050711 Bucharest, Romania; robertlunga95@gmail.com

**Keywords:** maladaptive schemas, violence, Punitiveness, Mistrust/Abuse, adolescents

## Abstract

This study aims to analyze maladaptive schemas through the Young Schema Questionnaire—Short Form 3 among 895 high school students, with an average age of 18.15 years, in relation to the potentially traumatic experience of being the victim of violence inflicted by family members through hitting and beating and in connection with violent behavior (in and outside school). Almost half of the students reported that, in their families, there were prolonged problems in the couple relationship of their parents/caregivers, and almost 40% of these students were involved from time to time in at least one form of violence in or outside school, with the highest share of this violence resulting from physical aggression by hitting and pushing and verbal or emotional abuse. A factor analysis was performed using a unifactorial model and a mediation model, and it indicated that the presence of trauma increases the total violence score. A higher violence score was recorded in students who were subjected to family violence (t(890) = −6.267, *p* < 0.001). The schemas that proved to be the most relevant for the violence factor were those of Punitiveness (PU: 0.89) and Mistrust/Abuse (MA: 0.77), followed by the schemas of Emotional Inhibition (EI: 0.68), Unrelenting Standards/Hypercriticalness (US: 0.63), and Entitlement/Grandiosity (ET: 0.58). The mediation that the Punitiveness schema achieves between victimization in the family and subsequent aggressive behavior is based on the internalization of the punitive parental figure and the victim’s development of the belief that violence is the only answer when others do not meet their expectations.

## 1. Introduction

Each year, approximately 193,000 homicides occur worldwide among individuals aged 15 to 29, representing 40% of all homicides (World Health Organization [WHO], 2024). Data on aggression and physical altercations collected in 40 developing countries indicate that, on average, 42% of boys and 37% of girls have experienced bullying (World Health Organization [WHO], 2024). Furthermore, the latest UNICEF statistics (UNICEF, 2024) reveal a concerning prevalence of sexual violence, with one in five girls and one in seven boys reporting sexual abuse.

In the national context, Romania shows similarly concerning trends: nearly 34% of 15-year-old students experienced bullying at least a few times per month, with disadvantaged school students exhibiting an even higher rate (39%); this percentage is significantly above the European average of 22.1% (European Commission: Directorate-General for Education, Youth, Sport and Culture, 2021).

The etiology of adolescent violent behavior is widely recognized as multifactorial, arising from a complex interplay of biological, genetic, psychological, and environmental (including social) factors (Klein & Fernàndez-Castillo, 2021; Ibrahim et al., 2024). Studies have highlighted associations between testosterone and/or cortisol levels and sensation seeking and reward sensitivity (Forbes et al., 2010), risk taking, and proactive or reactive aggression (Van Bokhoven et al., 2006; Calvete et al., 2025). However, researchers have expressed reservations about the predictive value of neurobiological markers for aggressive behavior, emphasizing the mediating role of environmental factors (Blankenstein et al., 2022; Pante et al., 2022).

During this stage of development, adolescents’ pursuit of autonomy and the establishment of new social and/or intimate relationships may become a source of frustration and conflicts. When these challenges are coupled with difficulties in emotional regulation, they can trigger the adoption of unhealthy coping strategies and associated internalizing problems like anxiety, depression, and withdrawal, as well as externalizing problems such as substance use, conduct issues, academic challenges, and juvenile delinquency (Çelik & Çalık, 2022; Gubbels et al., 2024).

Within the family domain, research has identified several factors, including family functioning and parenting styles, that serve as both critical protective and risk factors for violent and antisocial behavior in adolescents (Ciurbea et al., 2025). Protective factors such as secure attachment, warm and supportive parent–child relationships, and an authoritative parenting style—characterized by effective supervision, consistent discipline, reinforcement of positive behavior, disapproval of antisocial behavior, low physical punishment, clear norm orientation, and the child’s active participation in family life—may mitigate the risk of violence. Additionally, appropriate behavioral modeling by parents supports adolescents in developing nonviolent conflict resolution and emotional regulation skills (Lösel & Bender, 2017). Conversely, risk factors including lack of family cohesion and stability, insufficient warmth or emotional support, harsh or inconsistent discipline, poor supervision, low parental involvement in the child’s activities, exposure to family violence, marital conflict, and parent–child conflicts have been linked to increased aggression and antisocial behavior (Lansford et al., 2011; Eisner & Malti, 2015).

Several theories have been proposed to explain the association between exposure to violence and an increased risk of subsequent violent behavior, often through the hypothesis of the intergenerational transmission of violence. The social learning theory posited that children who are victims or witnesses of violence between parents or adult caregivers often internalize aggressive behavior as an acceptable method of solving conflict and are likely to replicate such patterns in their own interpersonal relationships (Bandura, 1977; Akers & Sellers, 2009). According to social information processing theories, a history of exposure to violence can lead to the development of biased perceptions of other’s actions by attributing them a hostile intent; the interpretation of ambiguous social contexts as threatening may result in the adoption of a belligerent strategy that, in turn, elicits aggressive responses from others—ultimately reinforcing violent behaviors (Dodge et al., 1990). Alternatively, the general theory of crime stipulates that children of dysfunctional caregivers often experience socialization deficits due to inadequate monitoring and discipline. As a result, they may develop low self-control, which is reflected in high impulsivity, a reduced capacity to consider the repercussions of their actions, and a propensity to seek out risky situations and engage in risky behaviors (Gottfredson & Hirschi, 1990).

Both child abuse and exposure to domestic violence are recognized forms of child maltreatment that, alongside other potentially traumatic adverse experiences with which they often coexist and intersect, are linked to a wide range of negative outcomes over the course of life. These include increased risks of depression, anxiety, anger, PTSD, psychosis, and addictive disorders (Gong & Chan, 2018; Sebalo et al., 2023; Vilariño et al., 2022). Moreover, these early adversities are associated with poor educational achievement, unemployment, poverty, and other negative socioeconomic trajectories (Merrick et al., 2018; Supol et al., 2021). Cumulative findings indicate revictimization and subsequent perpetration of violence as key consequences of early exposure to violence (Herrenkohl & Rousson, 2018; Widom & Maxfield, 2001). Adverse childhood experiences (ACEs) have been shown to predict risk behaviors not only in general adult populations but also among adolescents (Garrido et al., 2018). Specific forms of adversity—such as physical abuse, sexual abuse (whether occurring within or outside the family), parental substance use leading to household dysfunction, and interparental violence—are significantly associated with both interpersonal violence (e.g., delinquency, aggression, physical fights, dating violence, and weapon carrying at school) and self-directed violence (e.g., self-injury, suicidal ideation, and suicide attempts) during adolescence (Duke et al., 2010). Also, early experiences of victimization have been shown to be a stronger predictor of later involvement in violent behaviors than early involvement in violence (McAra & McVie, 2016).

Previous research has provided evidence for the association of physical, emotional, or sexual experiences of maltreatment in childhood with an increased risk of dating or intimate partner violence (Herbert et al., 2025; Kaukinen et al., 2015; Jennings et al., 2014). Exposure to family violence has also been linked to an increased likelihood of bullying perpetration among adolescents, although protective factors that restore adolescents’ sense of safety—such as strong school bonding—may buffer against this risk (Lawrence, 2022). Both cross-sectional and longitudinal studies have indicated that prior victims of child maltreatment abuse or neglect their own children (Berlin et al., 2011; Kong et al., 2021; Martoccio et al., 2022). Considering this established trajectory, early detection and intervention in cases of traumatic childhood adversities are critical for breaking the cycle of violence.

One therapeutic framework that provides valuable insight into the intergenerational transmission of violence—and offers guidance for targeted interventions—is schema therapy. This approach provides a fruitful explanation of how early adverse or unhealed traumatic experiences continue to shape key factors in the manifestation of aggression in adolescence, including emotional regulation, self-perception, and behavioral responses. Schema therapy also offers a structured pathway for addressing the deep-rooted vulnerabilities that may trigger or amplify violent behavior.

Schema therapy, which was developed by Young et al. (2003) as an extension of Beck’s (1979) cognitive–behavioral therapy, targets refractory cases involving relational problems (personality disorders or accentuated personality traits). Central to this theory is the early maladaptive schema (EMS), defined as “a broad, pervasive theme or pattern comprised of memories, emotions, cognitions, and bodily sensations regarding oneself and one’s relationships with others, developed during childhood or adolescence, elaborated throughout one’s lifetime and dysfunctional to a significant degree” (Young et al., 2003, p. 7).

The 18 schemas identified based on clinical experience were grouped into 5 domains corresponding to fundamental unmet needs: the need for secure attachment—Disconnection and Rejection domain with the schemas (1) Abandonment/Instability; (2) Mistrust/Abuse; (3) Emotional Deprivation; (4) Defectiveness/Shame; and (5) Social isolation/Alienation; the need for autonomy, competence, and a sense of identity—Impaired Autonomy and Performance domain with the schemas (6) Dependence/Incompetence; (7) Vulnerability to Harm or Illness; (8) Enmeshment/Undeveloped Self; and (9) Failure; the need for realistic limits and self-control—Impaired Limits domain with the schemas (10) Entitlement/Grandiosity and (11) Insufficient Self-Control and Self-Discipline; the need for the freedom to express valid needs and emotions—Other-Directedness domain with the schemas (12) Subjugation; (13) Self-Sacrifice; and (14) Approval-Seeking and Recognition-Seeking; and the need for spontaneity and playful engagement—Overvigilance and Inhibition domain with the schemas (15) Negativity/Pessimism; (16) Emotional Inhibition; (17) Unrelenting Standards/Hypercriticalness; and (18) Punitiveness.

The formation of maladaptive schemas can be attributed to four types of early life experiences: the frustration of basic needs, as derived from John Bowlby’s (1973) attachment theory; traumatization or victimization; a disruption in the balance between involvement and the respect for personal autonomy; and selective internalization or identification with significant individuals (Young et al., 2003, pp. 10–11). Typically crystallizing during childhood or adolescence, these schemas may intensify or ameliorate over the course of life. Maladaptive schemas can be unconditional (i.e., absolute beliefs about self and others that are formed early and accepted unconditionally) or conditional (i.e., later attempts to prevent negative consequences of the unconditional schemas). When a schema is activated by a new negative experience, it generates dysfunctional behavior depending on the adopted coping style (submissive, avoidant, or overcompensatory).

Although Jeffrey Young emphasized that early maladaptive schemas are most commonly formed in childhood as a result of the interplay between adverse experiences, emotional temperament, and cultural influences, he does not exclude the possibility that adolescence may also constitute a vulnerable period of development, in which they crystallize in response to new interpersonal traumas or unmet emotional needs: “They develop during childhood or adolescence and are elaborated throughout one’s lifetime” (Young et al., 2003, p. 61). Thus, during this period, previously acquired schemas are consolidated, or new ones are formed under the impact of new attachment disruptions, traumatic events, or noxious repeated experiences with cumulative effects. While maladaptive behaviors such as aggression may reinforce certain beliefs or coping strategies, they are better understood as expressions or consequences of existing schemas, rather than their origin. For example, in response to early experiences of emotional deprivation, vulnerability, or instability, a child could cope by adopting inflated self-beliefs or a sense of superiority to mask underlying feelings of insecurity or worthlessness, and this is how the Entitlement/Grandiosity schema may emerge. Violent or controlling behavior is not the cause of the schema but rather an expression or coping response to it. The perceived effectiveness of this behavior in achieving short-term goals (e.g., control, respect, or submission from others) can reinforce the overcompensation coping style and further entrench the schema.

A substantial body of literature has linked various adverse childhood experiences—such as maltreatment, insecure attachment, and authoritarian parenting or harsh disciplinary practices—to the development of early maladaptive schemas (Emami et al., 2024; Gong & Chan, 2018; Y. Wang et al., 2023). Pilkington et al.’s (2021) meta-analysis indicated, for example, among adults with a history of childhood abuse and neglect, the salience of schemas from the Disconnection and Rejection domain (notably the Emotional Deprivation, but also the Social Isolation, schema) and the Impaired Autonomy and Performance domain (particularly the Vulnerability to Harm or Illness schema). Similarly, May et al. (2022), in a meta-analysis focused on the adolescent population, reported strong associations between emotional neglect and several schemas within the Disconnection and Rejection domain (Abandonment/Instability, Mistrust/Abuse, and Social Isolation/Alienation), as well as the Impaired Autonomy and Performance domain (Failure). Emotional abuse, in contrast, was more strongly associated with the Emotional Deprivation schema from the Disconnection and Rejection domain and the Subjugation schema from the Other-Directedness domain. Further evidence is provided by Karantzas et al. (2023), whose meta-analysis highlighted significant positive associations between insecure attachment styles and early maladaptive schemas, with anxious attachment showing stronger links—particularly within the Disconnection and Rejection and Other-Directedness domains—compared to avoidant attachment.

Numerous studies have also established that early maladaptive schemas are associated with interpersonal problems–including abuse or violence victimization and abuse or violence perpetration–and a wide range of psychopathologies, such as anxiety, depression, eating, personality, and addictive disorders among both adults and adolescents (Janovsky et al., 2020; Zonnevijlle & Hildebrand, 2019).

Regarding violence, early maladaptive schemas were associated with teen and adult dating violence (Borges & Dell’Aglio, 2020; Shorey et al., 2017; Calvete et al., 2018b), intimate partner violence perpetration (Atmaca & Gençöz, 2016; Hassija et al., 2018; Rahme et al., 2025), cyber dating abuse (Celsi et al., 2021), and bullying perpetration (Yousefpour, 2022; Calvete et al., 2018a).

Although most research is cross-sectional, several longitudinal studies provide evidence for causal links between EMSs and violent behavior. These studies have shown that early maladaptive schemas can mediate the relationship between early adverse experiences and later aggression or victimization. For example, Calvete et al. (2015) found that low parental warmth in Year 1 predicted increased entitlement as well as Disconnection and Rejection early maladaptive schemas in Year 2, which in turn predicted child-to-parent aggression in Year 3. Similarly, Calvete et al. (2018b) demonstrated that early maladaptive schemas in the Disconnection and Rejection domain mediated the association between witnessing family violence and later dating violence victimization in girls. In another three-year longitudinal study, Calvete et al. (2018a) found that maladaptive schemas of Rejection mediated the predictive association between emotional abuse in the family and later victimization at school. Fernández-González et al. (2022) discovered that adolescent partner violence and child-to-parent aggression were not only correlated cross-sectionally but also longitudinally, mutually influencing one another over time. Additionally, maladaptive schemas exhibited varying predictive contributions regarding subsequent violence types: the Mistrust schema forecasted an escalation in dating violence perpetration, whereas the Grandiosity and Insufficient Self-Control schemas predicted an increase in child-to-parent violence.

### The Current Study

In recent years, although an increasing number of studies have explored the impact of these early maladaptive schemas and the psychosocial functioning of adolescents and have begun to demonstrate the utility of this theoretical framework in addressing behavioral or personality disorders, there is still insufficient data regarding the involvement of school students’ maladaptive schemas in violent behavior both at school and outside school.

This study aims to analyze maladaptive schemas in relation to the potentially traumatic experience of being a victim of violence inflicted by family members through hitting and beating and in connection with violent behavior, particularly in light of the worrying increase in the cases of violence among students.

Its novelty lies in complementing the specialized literature on violence within an understudied context, i.e., Eastern Europe, and, more specifically, Romania, with the goal of enhancing both the existing knowledge and psychological interventions necessary to combat school violence, given the limited efficacy of the current regulations.

The following two research questions were the focus of this study:

1. Is there an association between previous experiences of direct victimization in the family and the level of violence in school or outside school during adolescence?
2. Do maladaptive schemas, as proposed by schema therapy, mediate the relationship between adolescents’ previous experiences of direct familial victimization and their involvement in violent behavior in school or outside of school?

## 2. Methods

This study is part of a broader research project entitled “Protective and Risk Factors Regarding Youth Violence: A Psycho-Educational and Community Approach for Sustainable Development and Transformation”.

### 2.1. Assessment Instruments

Three psychological questionnaires were administered, standardized to the Romanian population: the Five-Factor Personality Questionnaire (CP5F) (Albu, 2008), the Strategic Approach to Coping Scale (SACS) (Budău & Albu, 2010), and the Young Schema Questionnaire—Short Form 3 (YSQ-S3) (Trip, 2006). Additionally, an omnibus questionnaire consisting of 41 items was used to collect, along with sociodemographic data, information on several important topics, such as family relationships, risky behaviors (early and unprotected sex, alcohol consumption, drug use, smoking, etc.), involvement in violent behavior both at and outside school, and others.

In this study, the responses obtained for the item regarding experiences in childhood and/or adolescence of being hit or beaten and on violent behavior in and outside school, as well as responses to the Young Schema Questionnaire—Short Form 3 (YSQ-S3), are analyzed.

School students were asked whether, during childhood or adolescence, they had been hit or beaten by their parents or other family members, with response options of “Of-ten”, “From time to time”, and “Never”. As the purpose of this research was to identify individuals who were exposed to family violence, this item was recoded into a dichotomous variable, Yes/No, by combining the “Often” and “From time to time” responses as “Yes”. Thus, an affirmative answer was taken as an indicator that the students had been victims of family violence through hitting or beating. Those who answered “Never” were classified as “No”, indicating no such experience. This recoding allowed for analyzing the prevalence and impact of family violence more straightforwardly. School students where asked whether, at or outside school, they had engaged in the following behaviors: (1) hit, pushed, or shoved someone when angry; (2) carried a knife, a sharp weapon, or another blade; (3) threatened someone with a knife or a sharp weapon; (4) verbally or emotionally abused someone or said something that made the person feel bad about themselves or instilled fear; (5) bullied or sexually assaulted someone; (6) robbed someone; and (7) gotten into a fight after drinking alcohol or using drugs. In addition, they were asked whether they had ever been suspended or expelled from school.

Furthermore, data collected through a series of questions regarding the experience of adolescents with significant, potentially traumatic life events within the family in which they were raised are analyzed (i.e., being separated from one of the parents/caregivers for more than a month, being subjected to harsh discipline, parental divorce, the migration for work of one or both parents/caregivers, alcohol dependency of one or both parents/caregivers, and the presence of conflicts in the couple relationship of the parents/caregivers).

### 2.2. Participants

In this study, 895 high school students, aged 18 and over, in their penultimate and final school years from 11 counties in Romania and six sectors of Bucharest were included.

Data collection took place from 15 January to 26 April 2024 and was conducted by specialist psychologists and school counselors. The inclusion criterion was being 18 years of age or older. The sample was based on convenience.

To ensure anonymity and maintain data confidentiality, the questionnaires were coded prior to administration by the school counselor (using the matriculation number, grade book number, or another code used by the investigator). Respondents signed an informed consent form alongside the assigned code.

As pilot tests measuring the completion time of the questionnaire set indicated an average of one hour and thirty minutes, the administration was carried out in two waves, with intervals of 1–3 weeks, to ensure that school students had up to 50 min to complete the questionnaires.

## 3. Results

### 3.1. Descriptive Data

The age of the school students ranged from 18 years (87.3%) to 22 years (0.1%) (mean: 18.15; median: 18; std. deviation: 0.416). The sample included 53% male school students and 47% female school students. In the analyzed sample, approximately 77% of school students reported engaging in violent behaviors, with 8% reporting very violent behaviors. This behavior was more frequently reported by male respondents (56%) compared to female respondents (46%).

**Figure 1:**
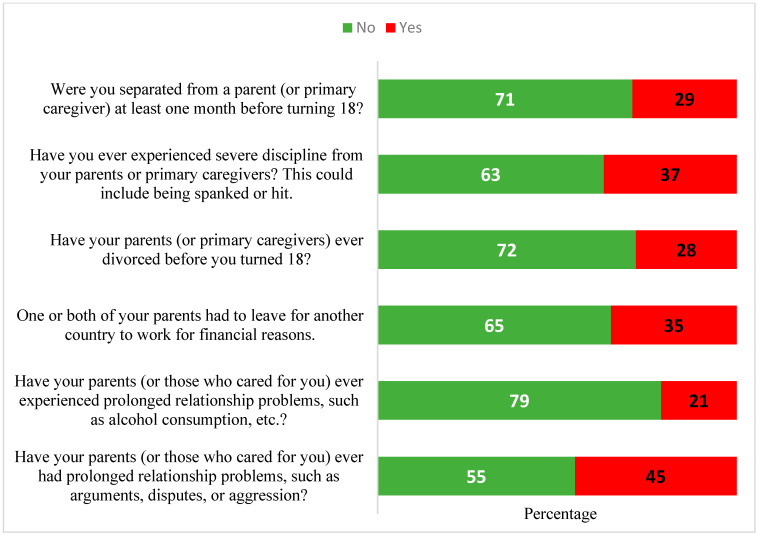
Percentages of school students reporting significant events in family life.

Almost half of the school students (45%) stated that there were prolonged issues in the couple relationships with their parents/caregivers. Regarding family separation, more than one-third of the students (35%) reported that one or both of their parents had gone to work abroad, and nearly one in three students (29%) had been separated from a parent/caregiver for extended periods. Additionally, almost one in three students (28%) came from a family in which the parents were divorced. One in five students (21%) witnessed alcohol dependency in one or both parents/caregivers. More than one-third of the students (37%) reported having experienced harsh acts of discipline (including spanking and beating) from their primary parents/caregivers (Figure 1).

Almost 40% of high school students have been involved from time to time in at least one form of violence in or outside school, with the highest share of this violence stemming from physical aggression through hitting and pushing and verbal or emotional abuse.

Internal consistency was measured in terms of Cronbach’s α, with Cronbach’s α = 0.743 reported for school violence and Cronbach’s α = 0.749 for outside-of-school violence. Violence scores were calculated as the averages of the associated item scores. The two scores correlated significantly, r (891) = 0.766, *p* < 0.01. The total score was computed as the sum of the scores for school violence and outside-of-school violence, and it was used further in the analysis. Descriptive statistics are shown in Table 1. Data were processed and analyzed using SPSS version 27 (IBM Corp., 2020).

The Young Cognitive Schemas Questionnaire—Short Form 3, consisting of 114 items, was used to measure, via scores, attitudes on self and one’s relationships with others. School students described themselves by rating the descriptive statements on a six-point Likert scale, ranging from “1 = completely untrue about me” to “6 = perfectly describes me”. Higher scores indicate a stronger presence for the respective schema.

The scores for the 18 dysfunctional cognitive schemas were calculated according to Young’s methodology. The 18 scores were reanalyzed through exploratory factor analysis (EFA) using JASP, version 0.19.3 (JASP Team, 2024). The EFA indicated that the most appropriate factorial model for the analyzed data is a two-factor model. Only cognitive schemas with correlations with the factors exceeding the reference value of 0.4 were considered of interest. The Kaiser–Meyer–Olkin test indicated MSA criterion values above 0.8 and an overall MSA of 0.949. The results of Bartlett’s Test of Sphericity were significant(*p* < 0.001), indicating that the factor analysis was justified.

Subsequent to the EFA, one dichotomous variable was used to identify individuals that reported violent behavior. Binary logistic regression was used to assess which of the EMS factor scores better predict violent behavior. For the first EMS factor, the analysis yielded an odds ratio (OR) of 1.54 for the high scores, with a 95% confidence interval (CI) of [1.16, 2.03], and *p* = 0.003. This indicated that for each additional unit of factor score, there is a 1.54 increase in the odds of developing violent behavior. For the second factor the odds ratio was not significant, OR = 1.01, *p* = 0.92. The Hosmer–Lemeshow test was conducted to assess the goodness-of-fit. The non-significant result (*p* = 0.91) indicated an acceptable fit of the model to the observed data. Consequently, only the first factor was retained for further analysis. The retained factor was associated with the following five maladaptive schemas: Mistrust/Abuse—MA; Emotional Inhibition—EI; Unrelenting Standards/Hypercriticalness—US; Entitlement/Grandiosity—ET; and Punitiveness—PU.

The internal consistency for the five scales, in descending order, was as follows: PU (α = 0.840, 14 items), MA (α = 0.772, 5 items), EI (α = 0.723, 5 items), US (α = 0.554, 5 items), and ET (α = 0.540, 5 items). The factorial score correlated significantly with the scores calculated for school violence (r = 0.146, *p* < 001) and outside-of-school violence (r = 0.197, *p* < 001). Bootstrap *t*-tests indicated significantly higher mean scores for the maladaptive schemas among those who had been victims of violence inflicted by family members through hitting or beating. MA scores in the victims of violence group (M = 16.91, SD = 5.66) compared to the no history of violence group (M = 15.56, SD = 5.91) were significantly higher, t(892) = −3.41, *p* < 0.001, and the Cohen’s d effect was d = −0.23. PU scores in the victims of violence group (M = 42.98, SD = 11.33) were also higher than those in the no history of violence group (M = 40.37, SD = 11.51), t(892) = −3.32, *p* < 0.001, and the Cohen’s d effect was d = −0.22. ET scores in the victims of violence group (M = 16.92, SD = 5.93) were higher compared to those in the no history of violence group (M = 15.58, SD = 4.92), t(892) = −3.65, *p* < 0.001. Cohen’s d effect was d = −0.25.

No significant differences were observed for EI and US scores. However, the scores in the family violence group were higher. This suggests that, despite the lack of overall differences, individuals exposed to family violence may have elevated levels in the measured areas, warranting further investigation into the implications of these findings.

### 3.2. Unifactorial Model

**Figure 2:**
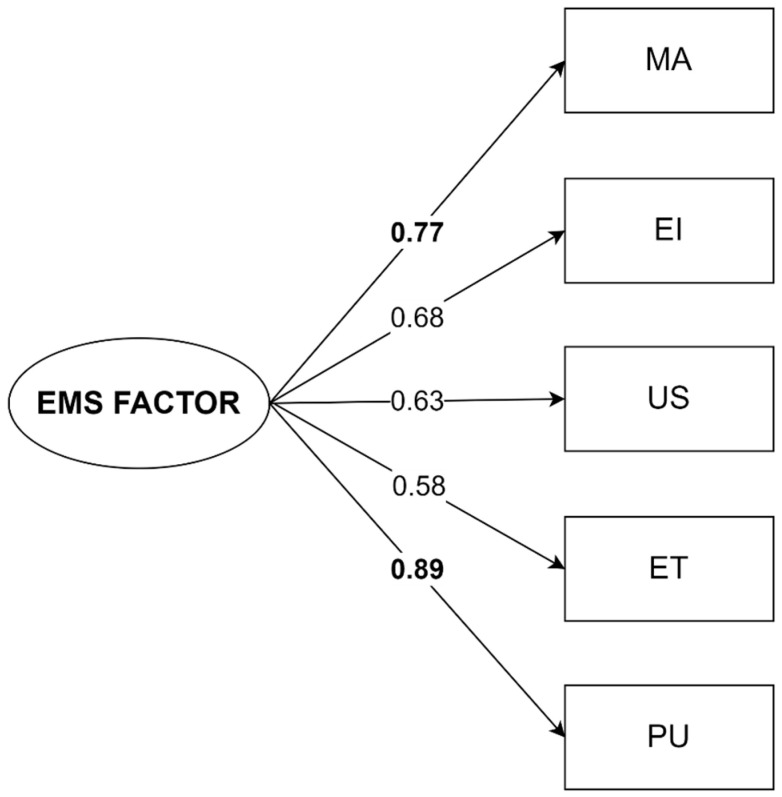
Unifactorial model. EMS Factor: placement for the maladaptive schema factor, MA: Mistrust/Abuse, EI: Emotional Inhibition, US: Unrelenting Standards/Hypercriticalness, ET: Entitlement/Grandiosity, and PU: Punitiveness.

This factor, with the potential to generate violent behavior, was reanalyzed using confirmatory factor analysis (CFA). The unifactorial model in CFA, presented in Figure 2, showed acceptable performance (CFI = 0.995; TLI = 0.997; RMSEA = 0.052; RMSEA *p*-value = 0.408). Internal consistency was acceptable, with Cronbach’s α = 0.807 (>0.8) and Average Variance Extracted (AVE) = 0.639 (>0.5). All correlations between the variables and the factor were positive, significant, and greater than 0.5. The most important correlations between the variables and the model’s factor (factor loadings) were for the PU schema (beta = 0.893) and the MA schema (beta = 0.770) (Figure 2).

Considering that the use of composite scores can offer a clearer and more concise picture of the relationships between variables, by facilitating the interpretation of results, as suggested by DiStefano et al. (2009), this study computed a factorial early maladaptive schema (EMS) score for analysis. The EMS score was retained for subsequent analyses as a composite variable, providing information on the respondent’s placement for this factor and offering a better understanding of how early maladaptive patterns manifest in behavior and thought.

### 3.3. Mediation Model

**Figure 3:**
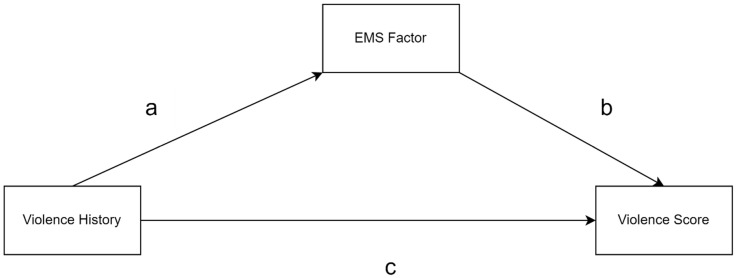
Conceptual mediation model of early maladaptive schema (EMS) factor score.

Subsequently, a mediation model was used in which being a victim of violence inflicted by family members through hitting or beating (violent treatment) was considered as a binary independent variable, while the total violence score, which is the sum of the school violence and outside-of-school violence scores, was considered as the dependent variable. The factorial EMS score (obtained through CFA) was used as the mediating variable. The conceptual diagram of the model is presented in Figure 3.

The unifactorial model indicated that those with a history of education through battering had higher mean scores on maladaptive schemas. The strongest correlations were observed for the Punitiveness (PU) and Mistrust/Abuse (MA) schemas.

The mediation model showed that being a victim of family violence (the trauma of violence) significantly influences the EMS factorial score (placement for the maladaptive schema factor), which in turn affects the PU and MA schemas. The estimated effect was positive, a = 1.0086, meaning that, for a school student with a history of family violence, the factorial score increases by 1.0086. Furthermore, the EMSD factor significantly influences the violence score. The estimated effect was positive, b = 0.0194. The indirect effect was ab = 0.0195. The standardized effects, β, and the bootstrap-calculated confidence intervals are presented in Table 2.

The direct effect between being a victim of violence inflicted by family members through hitting and beating (the trauma of violence) and the violence score was significantly positive, c = 0.1874. If trauma was present, it resulted in an increase in the total violence score. In addition, the *t*-test indicated a higher violence score among school students who experienced violence inflicted by their families (t(890) = −6.267, *p* < 001).

The schema correlation table below demonstrates significant correlations among all schemas. The strongest correlation was observed between MA and PU (r = 0.685, *p* < 0.001). All correlations are statistically significant at *p* < 0.001, indicating robust associations. The other three early maladaptive schemas, ET, EI, and US, associated with the latent schematic factor can also have the same mediation effect. The consistent, significant correlations imply that these schemas are interconnected. The particularly strong correlation between MA and PU suggests that punitiveness may play a mediating role or be influenced by maladaptive schemas, especially those related to entitlement and emotional deprivation. The data support the idea that MA, ET, EI, US, and PU can collectively serve as a latent factor, potentially mediating or moderating the relationships among violent behavior and a history of family violence, highlighting the complex interplay between individual schemas and behavioral outcomes (Table 3).

## 4. Discussions

The answer to the first research question is affirmative; specifically, previous experiences of family victimization are linked to the level of violence in school and outside school. The violent behavior score was higher for school students who experienced violence from their families. According to the data analysis of this study, based on 895 high school students with a mean age of 18.15 years, a significant correlation was identified between disciplinary practices, namely education through hitting and beating (family victimization), and an increased violence score (both in school and outside school), which suggests that experiences of family violence may be related to school students’ behaviors or attitudes related to violence. This finding is consistent with the literature, which indicates that a history of childhood abuse is associated with a greater risk of subsequent aggressiveness (Davis et al., 2018; Benedini et al., 2016; Milaniak & Widom, 2015).

Over time, research has increasingly acknowledged the cumulative impact of overlapping risk factors on child development. While the cycle of violence can be better understood through the lens of polyvictimization coupled with the absence of protective factors, family continues to play a crucial role—it can either set the pathway to violence or buffer the traumatic potential of other negative experiences by strengthening adolescents’ ability to cope with distress (Keels, 2022).

In Romania, support for corporal punishment remains high, particularly in environments where mentalities and practices continue to reflect the values of a traditional, patriarchal, and agrarian society. A study conducted by the Save the Children Organization (Organizația Salvați Copiii, 2021) on a nationally representative sample in Romania found a decrease in physical violence against children, from 38% in 2001 to 16% in 2021. This indicates that changes in legislation, social campaigns, and parenting programs have produced positive results. However, according to the “Child Well-being in Rural Romania” report by the World Vision Romania Foundation (2024), 2 out of 10 parents believed that it is sometimes necessary to beat their children to educate them better. This finding highlights that violent disciplinary practices still represent an issue in Romania and underscores the further need for educational intervention, as any justification for the use of violence in child-rearing or education is unacceptable.

This sociocultural context creates conditions for multiple forms of victimization within the family, as such family structures often exhibit a high tolerance for domestic violence. Within these settings, violence is frequently justified as a means of enforcing rigid, non-negotiable rules, with acts of aggression against women and children commonly framed as deserved punishment for the perceived breach of order. Additionally, the prevailing societal attitude tends to perpetuate a culture of victim-blaming while simultaneously minimizing or exonerating the responsibility of the aggressor.

Therefore, within an environment marked by overall familial aggression or instability, it’s difficult to draw a strict line between physical discipline and physical abuse. In numerous instances, children are subjected to physical punishment, either as a consequence of their own perceived transgression or for their mere presence during an adult’s moment of rage. In addition to direct victimization, they are frequently exposed to domestic violence, which can generate profound emotional consequences, including fear, helplessness, feelings of guilt for not intervening, and a sense of powerlessness in the face of ongoing abuse. An effective approach to raising and educating children requires a balance between authoritarian and permissive parenting styles, as well as the development of resilience and adaptive coping strategies (Panișoară, 2024), because, in any situation, educating or correcting through violence is not a solution.

Previous research in Romanian populations found that the highest incidence of all forms of violence against women, perpetrated by men within the family, is found among individuals who, during childhood or adolescence, were both witnesses to and victims of family violence in their family of origin (Rada, 2014).

The second research question of this study received an affirmative answer; namely, maladaptive schemas act as mediating factors in the relationship between previous experiences of direct victimization in the family and the level of violence at or outside school.

Maladaptive schemas had higher mean scores among those who reported being hit or beaten by parents or other family members during childhood or adolescence.

The composite factorial score of the early maladaptive schemas (EMSs) was influenced by the trauma of violence. The two main schemas that prove most relevant to the violence factor were Punitiveness (PU: 0.89) and Mistrust/Abuse (MA: 0.77), followed by the schemas of Emotional Inhibition (EI: 0.68), Unrelenting Standards/Hypercriticalness (US: 0.63), and Entitlement/Grandiosity (ET: 0.58).

Consequently, this study identified that violence was mediated by three schemas from the Overvigilance and Inhibition domain, one from the Disconnection and Rejection domain, and one from the Impaired Limits domain. Within the domains of Impaired Autonomy and Performance and Other-Directedness, no maladaptive schemas were found to be involved in violent behavior.

We expected that the Emotional Deprivation (ED) schema, which belongs to the Disconnection and Rejection domain, would be implicated in violent behavior. When a person’s emotional needs are unmet or when they feel deprived of the desired attention, understanding, or validation, they may develop feelings of entitlement or even aggressive behavior towards others (M. Wang et al., 2023; Arshad, 2024). A possible explanation is linked to the particular socialization conditions of Generation Z, who grew up with access to vast amounts of information and virtual interactions but demonstrate increased vulnerability to stress, anxiety, trauma, and depression when facing real-world challenges and managing frustrations (Haidt, 2024). Moreover, the Entitlement/Grandiosity (ET) schema identified in this study as part of the mediating factor between family violence and violence manifested at and outside school may serve as a form of overcompensation for the Emotional Deprivation (ED) schema.

Violence can also be a response to stress. The experimental study by Kruk et al. (2004) highlighted that stress and aggression can reinforce each other: the interaction between the body’s stress response and the brain’s aggression form a feedback loop that plays a significant role in regulating aggressive behavior. This is especially important when we consider chronic stress conditions, such as those created by economic hardship, residential instability, family conflicts, and major disasters like war or even a global public health crisis such as the COVID-19 pandemic. These circumstances can generate aggressive reactions among adolescents both directly and indirectly through the negative examples set by parents who respond violently to frustration.

Studies investigating the development of maladaptive schemas have focused primarily on emotional abuse and less on physical abuse (Calvete, 2014; McCarthy & Lumley, 2012; Wright et al., 2009). This study primarily targeted the physical component of aggression and only secondarily the emotional reverberation of victimization, although it must be acknowledged that any type of abuse is accompanied by an emotional component. Consequently, the findings of this study, despite being obtained within a cross-sectional design, can broaden our understanding of the role that family-of-origin victimization may play in perpetuating violence in other settings. In this regard, they complement the results reported by Calvete et al. (2018a) on family victimization as a risk factor for bullying. However, the research concentrated on the emotional component of both phenomena.

The belief that people should be harshly punished for their mistakes and intolerance toward those who fail to meet personal standards, without taking mitigating circumstances into account, as characterized by individuals with the Punitiveness schema (Young et al., 2003), can help explain the violent behavior observed both at school and outside among the school students in this study. Those who are treated in such a manner tend to treat others similarly.

The mediation that the Punitiveness schema exerts between family victimization and subsequent aggressive behavior is based on the internalization of a punitive parental figure. If, because of this identification, a person comes to believe that violence is a response (the only one) when others fail to meet their expectations, they may act in the manner they have learned. Moreover, it is equally plausible that the distress generated by the punitive figure may cause emotional dysregulation, leading to violent outbursts when the individual feels criticized or fails to meet certain expectations. It is also possible that aggression serves as a way to reenact a traumatic childhood situation. The practical implications of these findings emphasize the need to develop interventions aimed at helping adolescents with this schema to find and express their own voices, distinct from those of punitive parents, as well as to adopt strategies to resolve frustration that do not rely on aggression and control but rather on practicing the ability to forgive and show empathy.

The Punitiveness (PU) schema is an unconditional schema, which means that, according to Young, it was formed the earliest, occupies a central position, and encapsulates uncompromising beliefs about self and the world. If this schema formed as a result of physical corrections administered to a child, then the world becomes a place where being beaten because someone became upset or angry is considered an axiomatic truth. Subsequent attempts reduce the suffering produced by this schema and focus on ways in which the subject tries to avoid “deserving” punishment, without questioning the rigid manner in which any mistake, deviation, underachievement, or alteration from the expected outcome is treated. Therefore, the fact that the conditional schemas Unrelenting Standards/Hypercriticalness (US) (factor loading: 0.63) and Emotional Inhibition (EI) (factor loading: 0.68) follow the Punitiveness schema is entirely consistent with the principles outlined by Young et al. (2003) in differentiation schemas, as well as with their clinical observations regarding the PU–US tandem (p. 268).

The presence of both the US and Emotional Inhibition (EI) schemas indicates a rigidity in the value system, a consequence of internalizing external norms, rules, or expectations that have been imposed by force, which confirms that the school students in this study have internalized the suppression of emotional expression as a core value, as asserted by Young et al. (2003). This association between US and EI schemas can also be considered present among the investigated school students, as these are reflected in the composite schema score that mediates the relationship between the physical violence a child endures within the family and the violent behavior exhibited by the physically abused child. The result is relatively similar to that reported by Crawford and Wright (2007), who found that EI was involved in the relationship between psychological maltreatment and the perpetration of aggression and revictimization in interpersonal relationships during adulthood. They observed that the Emotional Inhibition schema clusters with the Mistrust/Abuse and Self-Sacrifice schemas to mediate the relationship between experiences of maltreatment and victimization, while together with the Entitlement/Grandiosity, Mistrust/Abuse, and Insufficient Self-Control and Self-Discipline schemas, it mediates the relationship between experiences of maltreatment and the perpetration of aggression against an intimate partner. In this study, due to the differing outcomes of these schema aggregations, the suppression of emotions is not a schema that dictates a specific course of action; on the contrary, the chosen coping strategies determine whether a subject acts violently, becomes a victim, or avoids confrontation. Moreover, the selection of these coping strategies is influenced by the presence of other cognitive schemas.

In a study analyzing the links between parenting practices and early maladaptive schemas, Gunty and Buri (2008) found that nearly half of the variation in these schemas could be attributed to specific parental influences. Specifically, of the variation in these two schemas, 33.8% was explained by paternal authoritarianism, 4.4% by parental psychological control, and 3.4% by maternal authoritarianism. Therefore, in accordance with the literature, it can be assumed that the development of schemas from the Overvigilance and Inhibition domain (of which this study identified three as being involved in violence) is significantly influenced by paternal authoritarianism.

The results of this study only partially confirm the central role of the Disconnection and Rejection domain in explaining aggressive behavior, namely through the Mistrust/Abuse schema. Considering the data reported in the literature, more schemas were expected to be identified from the Disconnection and Rejection domain among children raised with violence. Their absence may indicate either that the adaptive response to a lack of affection or the coping style to distress involves bypassing the Emotional Deprivation or Abandonment schemas, a result that cannot be countered in a self-report study, or it may be due to individuals overcompensating for these schemas through the display of emotional independence.

The contribution of the Mistrust/Abuse schema to the violence factor (factor loading: 0.77) is consistent with studies in the literature that have highlighted positive associations of this schema with anger (Calvete et al., 2005), aggression (Crawford & Wright, 2007; Dunne et al., 2018; Muris, 2006; Tremblay & Dozois, 2009), hostile intention attribution (Calvete & Orue, 2012), and sexual aggression (Sigre-Leirós et al., 2013).

The Mistrust/Abuse schema, formed as a result of repeated experiences of physical abuse perpetrated by one or more family members, leads the subject to expect to be abused, used, or humiliated by those around them and to develop anxiety, hypervigilance, and even paranoid ideation (Welburn et al., 2002). Being an unconditional schema, it indicates its primary nature and the difficulties in overcoming it, and it is associated with both revictimization and with transforming into an aggressor who abuses others (Young et al., 2003), depending on the configuration in which it is activated.

In a study that linked maladaptive schemas involved in aggressive behavior with social information processing, Calvete and Orue (2012) observed that, although the Mistrust schema is a predictor of inferences about the hostile intentions of others, it is also associated with emotional experiences other than anger (feelings of helplessness and sadness), which can inhibit the resorting to aggression to resolve conflicts and may lead to anxiety and depression. Additionally, Tremblay and Dozois (2009) recorded the strongest association of the Mistrust/Abuse schema with hostility, suggesting a conceptual overlap between suspicion and hostile attribution bias, i.e., the presence of erroneous perceptions regarding others’ intentions. These findings raise questions about the role played by this schema in triggering violent behavior. Based on the associations reported in the literature, this schema appears to represent a necessary, though not sufficient, cognitive distortion for aggression, at least in cases of unprovoked aggression.

Finally, the presence of the Entitlement/Grandiosity schema (factor loading: 0.63) in this study is consistent with that reported in the specialized literature, and it has a positive association with anger (Calvete et al., 2005; McKee et al., 2012), aggression (Crawford & Wright, 2007; Dunne et al., 2018; Gilbert et al., 2013; Tremblay & Dozois, 2009), and the attribution of hostile intentions and aggressive responses (Calvete & Orue, 2012). Typically, this schema forms due to an experience of extreme permissiveness and indulgence, with the subject growing up with the belief that the rules that apply to others do not apply to them due to a sense of superiority that must be maintained. These individuals often describe themselves as selfish (lacking respect or empathy for others) and impulsive (with zero tolerance for frustration and an inability to control their emotions or delay gratification). However, Young et al. (2003, p. 19) point out that Entitlement can also emerge as a form of overcompensation for the Emotional Deprivation and Defectiveness schemas. Consequently, in the present study, it is likely that overcompensation for an unmet emotional need is expressed through violent assertion.

Calvete and Orue (2010) advanced the hypothesis that, while the Grandiosity schema is directly linked to proactive aggression and, among the components of social information processing, is significantly associated only with anger, the Mistrust/Abuse schema might be more relevant to reactive aggression, being related to the interpretative component of social information processing. Reactive aggression is a response to a perceived threat or provocation (hence the importance of the erroneous attribution of hostile intentions) and is associated with negative affect and/or emotional lability, which in turn correlates with impulsive behavior; on the other hand, proactive aggression is related to the anticipation of reward, i.e., the expectation of a favorable outcome for aggression, and is associated with low emotionality, corresponding to calculated behavior (Zelli et al., 1999). Differentiating between these two forms based on the underlying cognitive and emotional processes is also important because early proactive aggression has been shown to be a predictor of subsequent delinquency, whereas early reactive aggression has been a predictor of violence in intimate relationships (Hubbard et al., 2010; Romero-Martinez et al., 2022).

The unique cluster in which the schemas identified as significant for the violence factor are found must be considered because the Mistrust/Abuse, Unrelenting Standards/Hypercriticalness, and Punitiveness schemas, along with the Self-Sacrifice schema, have been identified as having the greatest relevance in post-traumatic stress disorder (Bär et al., 2023). This implies that experiences similar to those of individuals who have suffered trauma can also be experienced and instilled in children who have been victimized within the family.

In conclusion, the findings of the present research support the mediating role of early maladaptive schemas in the relationship between previous experiences of direct family victimization and the level of violence at school or outside school, and they contribute to our understanding of how violence is perpetuated: once formed, these schemas directly contribute to an increased risk of subsequent violence by being activated in undesirable/problematic/difficult situations and the emergence, against the background of a dysfunctional coping style, of a schema mode that is relevant for violence (e.g., an angry child, an impulsive child, and a vulnerable child who overcompensates through anger (Edwards, 2022)).

From a practical standpoint, these findings underline the importance of early identification and psychological intervention in adolescents exposed to family violence. School-based mental health programs and violence-prevention initiatives could integrate schema-informed screening tools to detect maladaptive patterns in cognition and emotion regulation. Educators and school counselors could be trained to recognize signs of early maladaptive schema activation (e.g., disproportionate emotional responses, hostility, or social withdrawal) and refer students to appropriate services. Interventions such as schema therapy-adapted techniques, trauma-focused Cognitive Behavioral Therapy, or emotion regulation training could help young people reframe maladaptive beliefs, develop healthier coping styles, and reduce the risk of violent behavior.

## 5. Conclusions

Adolescents who experienced family victimization tend to register higher violence scores. Reports of physical aggression (e.g., beatings or hitting by family members) are also associated with elevated schema scores, particularly the Punitiveness (PU) and Mistrust/Abuse (MA) schemas. Furthermore, adolescents with higher maladaptive schema scores also tend to exhibit greater involvement in violent behavior, both within and outside school contexts. Within schema theory, this mediating relationship may reflect the possible contribution of the PU and MA schemas to a heightened state of vigilance, where the individual remains constantly alert and primed to respond to perceived threats, whether real or imagined; even more deeply, this may be an expression of the Vulnerable Child Mode and the Angry Child Mode that arose from unmet basic emotional needs (Aalbers et al., 2021).

Obviously, a child who has been beaten does not have his needs for affection, acceptance, competence, a sense of identity, and the freedom to express opinions met, and, consequently, finds it difficult to access a Happy Child Mode or a Healthy Adult Mode.

Reducing the perpetuation of violence in the general population, and particularly among adolescents and young adults, must be viewed as a three-phase process addressing their past, present, and future. Although the past cannot be changed, certain aspects of one’s life history can be addressed through schema therapy to overcome traumatic situations stemming from the family of origin, such as being educated through beating, as identified in this study.

Considering that this study identified that three schemas from the Overvigilance and Inhibition domain (Emotional Inhibition (EI), Unrelenting Standards/Hypercriticalness (US), and Punitiveness (PU)) that mediated violence (beating and hitting in the family and violent behavior at and outside school), there is a clear need for measures aimed at overcoming these ways of thinking that were formed as a result of victimization in childhood.

Therefore, two broad categories of measures are required to change these schemas formed as a result of childhood victimization.

The first aims at the correct identification and psychotherapeutic approach of the dysfunctional schemas and schema modes present in aggressive subjects. The involvement of the Mistrust/Abuse (MA) schema from the Disconnection and Rejection domain and Entitlement/Grandiosity (ET) schema from the Impaired Limits domain in mediating the two types of violence should not be overlooked, even though each domain is represented by only one mediating schema. Enhancing the ability to form trusting relationships and to accept realistic limits both for self and for others would help to overcome these two cognitive schemas, enabling individuals to view the world and life through a lens different from that of their parents, who, despite intending to educate, relied on violence as a means of discipline. Clinical experience has shown that individuals with a pronounced punitive parent mode have experienced one or more forms of childhood abuse, making it essential to interrupt this vicious cycle of violence.

Starting in 2024, Romania has had a National Plan with which to combat school violence, which includes procedures for teachers, teacher training, and responsibilities of schools and school inspectorates when violent incidents occur. While these measures are expected to produce results, they are insufficient. Interventions such as counseling and psychotherapy are required to overcome the negative patterns formed in school students within the family because of aggressive treatment. Early intervention would yield faster results, as it would not allow maladaptive schemas time to consolidate, considering that today’s adolescents will become employees, partners in relationships, and parents in the future.

The second aims at preventing the formation of these schemas by promoting non-violent educational methods. Both parents and other adults involved in the formal and informal education of children must be discouraged from using physical punishment, verbal violence, or humiliating treatments to discipline them. These adults must in turn receive guidance in order to be able to correct problematic behaviors and manage emotional crises. The ineffectiveness of physically punishing children should be emphasized in educational programs because this approach will not teach them that their behavior is wrong; rather, it may cause them to develop maladaptive cognitive schemas regarding communication and interpersonal relationships.

This study has practical relevance because it indicates that, while schemas can be grouped into certain domains, other clusters may also form that signal a thinking pattern predisposed to violent behavior.

At least one of the schemas identified in this study as being involved in the violent behavior of high school students was also found to be implicated in substance abuse, including drug and alcohol use (Vieira et al., 2023). Consequently, this study may have predictive value regarding substance use.

### Limitations

The main limitation of this study is its lack of national representativeness and the reliance on self-reported data.

Further studies are needed to confirm and refine this study’s results regarding the Punitiveness schema. Currently, we cannot determine whether this intolerance for mistakes and the inability to forgive are directed solely towards others or also towards self. The application of the revised schema questionnaire (YSQ-R) (Yalcin et al., 2023) would address this shortcoming and provide additional data in this regard.

More studies are also necessary to clarify why schemas from the Impaired Autonomy and Performance and Other-Directedness domains were not identified as mediators in the relationship between a history of childhood/adolescent family violence and violent behavior at and outside school among high school seniors.

Research on the formation of maladaptive schemas due to family violence victimization, which can subsequently generate violent behavior in adolescents and young adults, should also examine the modes in schema therapy (Yakın & Arntz, 2023). This approach could help reduce the activation of maladaptive schemas in young people by fostering the development of a Healthy Adult Mode in two key areas: increasing the ability to step back and reflect and enhancing emotional connection and tolerance.

## Figures and Tables

**Table 1:** Descriptive statistics.

Score	N	Minimum	Maximum	Mean	Std. Deviation
Violence in school	895	0.00	2.00	0.1933	0.24505
Violence outside school	893	0.00	2.00	0.2383	0.27813
Valid N (listwise)	893				

**Table 2:** Model effects.

				95% C.I.				
Indirect and Total Effects		Estimate	SE	Lower	Upper	β	z	*p*
Indirect	ab	0.019	0.006	0.007	0.032	0.019	2.90	0.004
Component	a	1.008	0.2855	0.474	1.526	0.117	3.54	<0.001
	b	0.019	0.003	0.010	0.026	0.165	5.08	<0.001
Direct	c	0.187	0.032	0.117	0.261	0.186	5.72	<0.001
Total	ab + c	0.206	0.032	0.1353	0.276	0.2056	6.27	<0.001

Note: Confidence intervals were computed with the bootstrap percentile method. Betas are completely standardized effect sizes.

**Table 3:** Schema correlation table (*n* = 985).

			Pearson			Spearman		
			r		*p*	rho		*p*
MA	-	EI	0.521	***	<0.001	0.496	***	<0.001
MA	-	US	0.466	***	<0.001	0.438	***	<0.001
MA	-	ET	0.491	***	<0.001	0.513	***	<0.001
MA	-	PU	0.685	***	<0.001	0.670	***	<0.001
EI	-	US	0.420	***	<0.001	0.386	***	<0.001
EI	-	ET	0.370	***	<0.001	0.391	***	<0.001
EI	-	PU	0.613	***	<0.001	0.586	***	<0.001
US	-	ET	0.516	***	<0.001	0.523	***	<0.001
US	-	PU	0.567	***	<0.001	0.520	***	<0.001
ET	-	PU	0.503	***	<0.001	0.524	***	<0.001

*** *p* < 0.001.

## Data Availability

The database supporting the reported results is available only upon request due to confidentiality and ethical restrictions.
